# GLP-1 receptor agonists in kidney transplant recipients with post-transplant diabetes: efficacy and safety outcomes from a retrospective cohort study

**DOI:** 10.20945/2359-4292-2026-0076

**Published:** 2026-07-22

**Authors:** Ricardo E. T. Navarrete, Joana C. Freitas, Isabel Fonseca, Ana Cunha, La Salete Martins, Joao Roberto Sa

**Affiliations:** 1 Programa de Doutorado em Medicina - Faculdade de Medicina da Universidade do Porto, Porto, Portugal; 2 Departamento de Endocrinologia e Metabologia, Hospital Beneficência Portuguesa, São Paulo, SP, Brasil; 3 Serviço de Nefrologia e Transplante Renal, Centro Hospitalar Universitário de Santo António, Porto, Portugal; 4 Divisão de Endocrinologia, Centro Universitário Faculdade de Medicina do ABC, Santo André, SP, Brasil

**Keywords:** kidney transplantation, diabetes mellitus, GLP-1 receptor agonists, glycemic control, renal outcomes, safety

## Abstract

**Objective:**

To assess the efficacy and safety of glucagon-like peptide-1 receptor agonists (GLP-1 RAs) in kidney transplant recipients (KTRs) with post-transplant diabetes mellitus (PTDM).

**Subjects and methods:**

This retrospective, single-center cohort study included 24 KTRs with PTDM who initiated GLP-1 RA therapy as an add-on to existing treatments between August 2013 and April 2024. Outcomes assessed included fasting plasma glucose (FPG), glycated hemoglobin (HbA1c), body weight, body mass index (BMI), blood pressure, lipid profile, renal function, and adverse events, with data collected at baseline and last follow-up.

**Results:**

Over a mean follow-up of 3.2 ± 2.1 years, GLP-1 RA therapy was associated with trends toward weight loss (-3.6 ± 8.5 kg; *p* = 0.051) and BMI reduction (-1.4 ± 3.3 kg/m^2^; *p* = 0.056). HbA1c decreased by -0.2% (*p* = 0.362), while FPG increased by +12.1 mg/dL (*p* = 0.232). Significant improvements were observed in total cholesterol (-41.5 mg/dL; *p* < 0.001) and systolic blood pressure (-8.5 mmHg; *p* = 0.041). Serum creatinine and estimated glomerular filtration rate remained stable (-0.09 mg/dL, *p* = 0.305; +3.2 mL/min/1.73 m^2^, *p* = 0.206, respectively). Nausea occurred in 21% (n = 5), and no other severe adverse events were reported.

**Conclusion:**

In KTRs with PTDM, GLP-1 RA therapy was associated with improvements in lipid profile and blood pressure, accompanied by non-significant trends toward weight reduction and stable renal function, and demonstrated an acceptable safety profile. Prospective studies are warranted to confirm long-term cardiorenal benefits.

## INTRODUCTION

Post-transplant diabetes mellitus (PTDM) is a common and clinically significant metabolic complication following kidney transplantation. Its pathogenesis is complex and multifactorial, frequently representing the progression of a dysmetabolic continuum that may begin prior to transplantation (^1^). This condition compromises both patient survival and long-term graft function. Key contributing factors include pre-transplant overweight or obesity and excessive post-transplant weight gain, both of which exacerbate insulin resistance and accelerate pancreatic β-cell dysfunction (^2^-^4^). Moreover, the combination of immunosuppressive therapy-particularly glucocorticoids and calcineurin inhibitors-alongside surgical stress and shifting metabolic demands creates a physiologically unstable environment that predisposes patients to early post-transplant hyperglycemia (^5^).

Despite the high prevalence and clinical burden of PTDM, therapeutic options for its management remain limited, especially in consideration of graft function and the complexities of immunosuppressive regimens (^6^). Traditional agents such as sulfonylureas are commonly used in this setting but carry an increased risk of hypoglycemia, particularly in patients with impaired renal function. Repaglinide has shown modest efficacy in transplant recipients with PTDM, while dipeptidyl peptidase-4 inhibitors have demonstrated improvements in insulin resistance and β-cell function without increasing the risk of hypoglycemia. However, these inhibitors have neutral cardiovascular effects, and their pharmacokinetic interactions with immunosuppressants may require closer monitoring of immunosuppressive drug levels (^7^,^8^).

In this context, glucagon-like peptide-1 receptor agonists (GLP-1 RAs) have emerged as promising therapeutic agents. These agents are structurally analogous to endogenous GLP-1, underpinning their metabolic efficacy. In type 2 diabetes, GLP-1 RAs are well established as effective therapies, achieving significant HbA1c reductions, sustained weight loss, and a low risk of hypoglycemia; several agents are also approved for obesity due to their favorable effects on body weight and glycemic control. Their pleiotropic actions extend beyond glycemic control and include appetite suppression, delayed gastric emptying, enhanced glucose-dependent insulin secretion, and improved insulin sensitivity. In the kidney transplantation, where post-transplant weight gain is frequent and metabolic risk factors are highly prevalent, GLP-1 RAs offer the advantage of addressing both hyperglycemia and excess weight, while minimizing hypoglycemia risk. This is a critical consideration in transplant recipients exposed to complex immunosuppressive regimens and multiple comorbidities. This dual benefit supports their potential role in individualized metabolic management for this high cardiometabolic risk (^9^,^10^).

Large cardiovascular outcomes trials, such as LEADER (^11^), SUSTAIN-6 (^12^), PIONEER 6 (^13^), and REWIND (^14^), have demonstrated reductions in major adverse cardiovascular events (MACEs) and renoprotective effects, establishing GLP-1 RAs as recommended treatment options for individuals with diabetes and elevated cardiorenal risk. These benefits have led to their prioritization in contemporary clinical practice guidelines, including for patients with chronic kidney disease or heart failure, irrespective of glycemic targets (^15^).

Nevertheless, robust data on the use of GLP-1 RAs in KTRs are still lacking, despite the clinical need for effective and safe therapeutic strategies in this setting. These patients often exhibit overlapping metabolic, cardiovascular, and immunosuppressive challenges, necessitating interventions that address not only glycemic control but also long-term risk modification. In this retrospective study, we examined the metabolic impact, safety, and treatment patterns of GLP-1 RA therapy in kidney transplant recipients with PTDM.

## SUBJECTS AND METHODS

This retrospective, single-center cohort study was conducted at a tertiary university hospital kidney transplant center. Eligible participants were KTRs aged > 18 years with a diagnosis of PTDM who initiated a GLP-1 RA as an add-on to existing antidiabetic regimens. General exclusion criteria were multi-organ transplantation, pre-existing diabetes (type 2 or other forms), and missing baseline or follow-up laboratory data. The specific exclusion criterion was concomitant sodium-glucose cotransporter 2 inhibitors (SGLT2i) therapy, in order to avoid confounding.

The study (reference no. 2022.264 [209-DEFI/224-CE]) was approved by the institutional Human Research Ethics Committee, with informed consent waived due to the retrospective and anonymized data collection. Patients initiating GLP-1 RA therapy between August 2013 and April 2024 were eligible for inclusion. Follow-up continued until treatment discontinuation, switch to another GLP-1 RA, or the study cutoff date. Inclusion in outcome analyses required at least one follow-up visit and a minimum of three months of continuous therapy.

### Data collection

Data collected included patient demographics (e.g., age, sex, weight, body mass index [BMI]), pre-transplant comorbidities, immunosuppressive regimens, and PTDM-related information. Treatment data comprised the specific GLP-1 RA agent, initial and maintenance doses, and concomitant medications (antihypertensives, lipid-lowering agents, and antidiabetic therapies), which were recorded at GLP-1 RA initiation and at the last follow-up. Clinical and laboratory variables assessed at baseline and follow-up included hemodynamic measures (systolic blood pressure [SBP] and diastolic blood pressure [DBP]), glycemic indices (fasting plasma glucose [FPG], HbA1c), lipid parameters (total cholesterol, low-density lipoprotein cholesterol [LDL-c], high-density lipoprotein cholesterol [HDL-c], triglycerides), and renal function markers (urea, serum creatinine, estimated glomerular filtration rate [eGFR]). Safety evaluation included adverse events potentially related to GLP-1 RA therapy: gastrointestinal symptoms, hypoglycemia, diabetic ketoacidosis, volume depletion, hospitalizations, and treatment discontinuation. **Table 1** summarizes the diagnostic criteria for PTDM, eGFR, BMI, and relevant comorbidities, with all parameters standardized according to established guidelines to ensure analytical consistency (^1^,^16^-^19^).

**Table 1:** Clinical definitions and diagnostic criteria used in this study

Parameter	Definition	Source
PTDM	HbA1c ≥6.5% or FPG ≥126 mg/dL ≥ 3 months post-transplant	ADA (^16^) Sharif et al. (^1^)
eGFR	2021 CKD-EPI equation	NKF-K/DOQI (^17^)
BMI	Weight (kg)/height (m^2^)	WHO (^18^)
Dyslipidemia	Abnormal lipid levels or use of lipid-lowering therapy	KDIGO-CKD (^20^)
Obesity	BMI ≥ 30 kg/m^2^	WHO (^18^)
Hypertension	BP ≥ 130/80 mmHg or antihypertensive use	NKF-K/DOQI (^17^)
Anemia	Hemoglobin < 11 g/dL (men/women)	KDIGO-CKD (^19^)

ADA: American Diabetes Association; BMI: body mass index; BP: blood pressure; CKD-EPI: chronic kidney disease epidemiology collaboration; eGFR: estimated glomerular filtration rate; FPG: fasting plasma glucose; HbA1c: glycated hemoglobin; NKF-K/DOQI: National Kidney Foundation Kidney Disease Outcomes Quality Initiative; PTDM: post-transplant diabetes mellitus; WHO: World Health Organization.

GLP-1 RA: Glucagon-like peptide-1 receptor agonists; SGLT2i: sodium-glucose cotransporter-2 inhibitors; PTDM: post-transplant diabetes mellitus; KT: kidney transplantation.

### Outcome measures

Efficacy outcomes included changes in FPG, HbA1c, total cholesterol, LDL-c, HDL-c, triglycerides, BMI, SBP, and DBP. Safety outcomes focused on renal parameters (eGFR and serum creatinine) and therapy-related adverse events, specifically hypoglycemia, gastrointestinal intolerance, and other drug-related events. All laboratory tests were conducted using standardized methods at the institution’s central laboratory.

### Statistical analysis

Data management was performed in Microsoft Excel (v. 2108), and analyses were conducted using SAS (v. 9.4; SAS Institute Inc., USA). Continuous variables are reported as mean ± standard deviation or median with interquartile range (Q1-Q3), depending on distribution as assessed by the Shapiro-Wilk test. Categorical variables are presented as absolute frequencies and percentages. Within-group comparisons between baseline and follow-up were assessed using paired Student’s t-tests for normally distributed variables and Wilcoxon signed-rank tests for non-normally distributed data. For subgroup analyses, independent t-tests or Mann-Whitney U tests were used as appropriate. A two-sided *p*-value < 0.05 was considered statistically significant.

## RESULTS

### Derivation of the cohort

**Figure 1:**
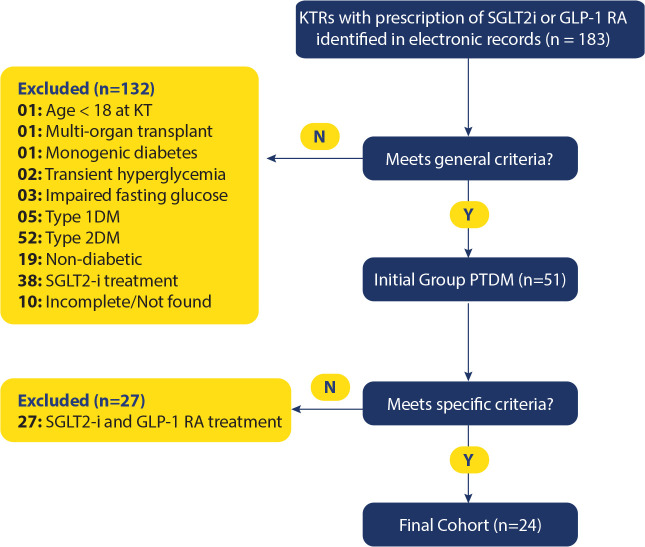
Flowchart depicting the cohort selection.

Of 183 KTRs initially screened, 132 were excluded for the following criteria: age <18 years (n = 1), multi-organ transplantation (n = 1), monogenic diabetes (n = 1), transient hyperglycemia (n = 2), impaired fasting glucose (n = 3), pre-existing type 1 (n = 5) or type 2 diabetes (n = 52), non-diabetic status (n = 19), incomplete data (n = 10), or initiation of SGLT2i as add-on therapy (n = 38). The remaining 51 patients had confirmed PTDM and were treated with either GLP-1 RA or combined GLP-1 RA and SGLT2 inhibitors. Of these, 27 patients receiving combination therapy were excluded to avoid treatment confounding. The final cohort consisted of 24 patients who initiated GLP-1 RA as add-on therapy to their existing antidiabetic regimen (**Figure 1**).

### Baseline characteristics

The cohort included 24 KTRs with PTDM, predominantly male (67%), with a mean age of 61.5 ± 10.3 years. All patients were White. Hypertension and dyslipidemia were universal, whereas obesity (BMI ≥ 30 kg/m^2^) was present in 29%. Heart failure and ischemic heart disease were documented in 46% and 21% of patients, respectively. Most grafts were from deceased donors (75%), and the majority were first transplants (92%). Immunosuppressive therapy primarily comprised corticosteroids (96%), mycophenolate mofetil (92%), and tacrolimus (83%). The mean duration of PTDM was 5.7 ± 3.5 years, and the mean time from transplantation to GLP-1 RA initiation was 7.6 ± 4.9 years. At baseline, mean HbA1c was 7.4 ± 1.7%, BMI was 27.3 ± 5.4 kg/m^2^, and eGFR was 46.1 ± 15.7 mL/min/1.73 m^2^. The lipid profile showed elevated triglycerides (200.5 ± 126.9 mg/dL) and LDL-c (94.9 ± 51.7 mg/dL). GLP-1 RAs were initiated as add-on therapy in all patients: dulaglutide (50%), liraglutide (25%), and semaglutide (25%), with a mean treatment duration of 3.2 ± 2.1 years. Detailed baseline characteristics are presented in **Table 2.**

**Table 2:** Baseline characteristics of KTRs with PTDM treated with GLP-1 RAs

Variables	Value
	mean ± SD (range) or n (%)
Demographic characteristics Age (years) Sex (male/female) Ethnicity (white) Family history of diabetes Comorbidities Hypertension Dyslipidemia Hyperuricemia Obesity (based on BMI ≥ 30 kg/m^2^) Heart failure Ischemic heart disease	61.5 ± 10.3 (46.0-81.0) 16 (67)/8 (33) 24 (100) 11 (46) 24 (100) 24 (100) 9 (38) 7 (29) 11 (46) 5 (^21^)
Preand post-transplant clinical history Time from KT to study inclusion (years) Primary kidney disease Glomerular diseases Tubulointerstitial diseases ADPKD Unknown Other causes	7.6 ± 4.9 (1.6-18.0) 6 (^25^) 4 (^17^) 4 (^17^) 8 (33) 2 (^8^)
Duration of renal replacement therapy (years) Hemodialysis/peritoneal dialysis/none Hepatitis C status Cytomegalovirus serostatus Donor type (deceased/living) Transplant number (first/retransplant) Immunosuppressive agents Corticosteroids Mycophenolate mofetil mTOR inhibitors Tacrolimus Cyclosporine Azathioprine	5.6 ± 5.7 (1.0-21.3) 18 (75) / 6 (^25^)/NR NR 14 (58) 18 (75)/6 (^25^) 22 (92)/2 (^8^) 23 (96) 22 (92) 20 (83) 5 (^21^) 3 (^12^) 2 (^8^)
PTDM-Related data Duration of PTDM (years) Duration of GLP-1 RA therapy (years)	5.7 ± 3.5 (0.3-10.6) 3.2 ± 2.1 (1.4-6.3)
Laboratory parameters Hemoglobin (g/L) HbA1c (%) Triglycerides (mg/dL) Serum creatinine (mg/dL) eGFR (mL/min/1.73 m^2^)	12.5 ± 1.7 (9.9-16.1) 7.4 ± 1.7 (5.7-11.5) 200.5 ± 126.9 (51.0-586.0) 1.7 ± 0.6 (1.0-2.7) 46.1 ± 15.8 (27.0-85.6)
Antihypertensive and lipid-lowering therapies Statins Ezetimibe (all in combination with statins) Angiotensin-converting enzyme inhibitors Angiotensin II receptor blockers Calcium channel blockers Beta-blockers Diuretics	24 (100) 10 (42) 14 (58) 5 (^21^) 16 (67) 15 (62) 7 (29)

ADPKD: autosomal dominant polycystic kidney disease; BMI: body mass index; eGFR: estimated glomerular filtration rate; GLP-1 RA: glucagon-like peptide-1 receptor agonists; HbA1c: glycated hemoglobin; KT: kidney transplantation; NR: not reported; PTDM: post-transplant diabetes mellitus.

### Changes in antidiabetic therapy

Following GLP-1 RA initiation, adjustments in background antidiabetic therapy were observed. Sulfonylurea use decreased from 17% to 8%, and DPP-4 inhibitor use declined from 29% to 17%, while metformin use decreased from 29% to 25%. Among GLP-1 RAs, dosing varied as follows: dulaglutide (0.75 mg [33%] or 1.5 mg [67%] weekly), semaglutide (0.25 mg [17%] or 1.0 mg [83%] weekly), and liraglutide (1.2 mg [50%] or 1.8 mg [50%] daily). Treatment discontinuation occurred in four patients (17%), all due to gastrointestinal intolerance, predominantly nausea. Basal insulin use increased from 46% at baseline to 66% at final evaluation. Details regarding GLP-1 RA type, dosing, and discontinuation are listed in **Table 3**.

**Table 3:** GLP-1 RA Regimens, treatment duration, and adverse events

GLP-1 RA	n (%)	Dose: n (%)	Mean duration (years)	Discontinuation, n (%)	Main adverse events
Dulaglutide	12 (50)	0.75 mg: 4 (33); 1.5 mg: 8 (67)	3.4 ± 2.1	2/12 (^17^)	Nausea
Liraglutide	6 (^25^)	1.2 mg: 3 (50); 1.8 mg: 3 (50)	3.1 ± 1.9	0 (0)	None reported
Semaglutide	6 (^25^)	0.25 mg: 1 (^17^); 1.0 mg: 5 (83)	3.0 ± 2.2	2/6 (33)	Nausea
Total	24 (100)		3.2 ± 2.1	4/24 (^17^)	

### Efficacy outcomes

**Figure 2:**
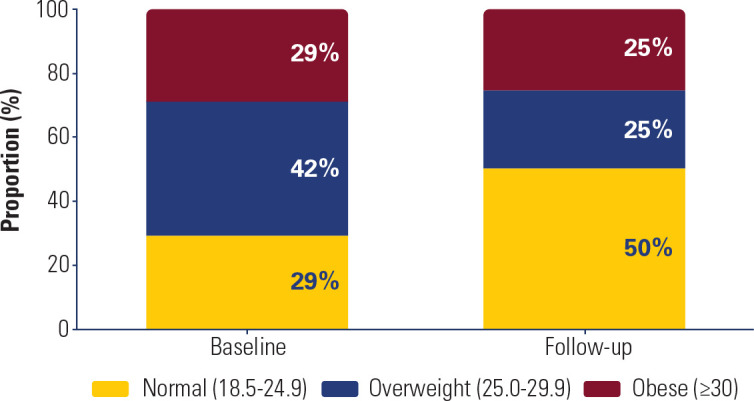
Change in BMI category distribution from baseline to follow-up during GLP-1 RA treatment.

Glycemic control remained stable, with HbA1c showing a non-significant reduction from 7.4 ± 1.7% to 7.2 ± 1.2% (*p* = 0.362), and FPG increasing from 114.5 ± 29.8 to 126.5 ± 37.9 mg/dL (*p* = 0.232). Significant improvements were observed in total cholesterol (182.9 ± 61.9 to 141.4 ± 32.6 mg/dL; *p* < 0.001) and LDL-c (94.9 ± 51.7 to 72.4 ± 28.6 mg/dL; *p* = 0.014). Triglycerides and HDL-c remained unchanged (*p* = 0.149 and *p* = 0.399, respectively). No adjustments to statin therapy were made during follow-up. Anthropometric parameters showed trends toward improvement that did not reach statistical significance: body weight decreased from 73.2 ± 14.7 to 69.6 ± 14.0 kg (*p* = 0.051) and BMI from 27.3 ± 5.4 to 25.9 ± 5.2 kg/m^2^ (*p* = 0.056). The proportion of normal-weight patients increased from 29% to 50%, while obesity prevalence decreased from 29% to 25% (**Figure 2**). The SBP decreased significantly from 143.0 ± 15.7 to 134.5 ± 15.6 mmHg (*p* = 0.041), while DBP remained stable (*p* = 0.665). These changes occurred without the introduction of new antihypertensive drug classes or escalation to higher-potency agents. Detailed results are reported in **Table 3**.

### Safety outcomes

Renal function remained stable throughout the follow-up period, with no significant changes in serum creatinine (1.7 ± 0.6 to 1.6 ± 0.6 mg/dL; *p* = 0.305) or eGFR (46.1 ± 15.8 to 49.3 ± 14.7 mL/min/1.73 m^2^; *p* = 0.206) (**Table 4**). Among 20 patients on tacrolimus-based immunosuppression with available data, mean tacrolimus trough levels remained stable from baseline to final follow-up (6.4 ± 2.1 vs. 6.2 ± 2.3 ng/mL; *p* = 0.68). Gastrointestinal intolerance was the most frequent adverse event, with nausea reported in five patients (21%), leading to treatment discontinuation in four cases (two on dulaglutide, two on semaglutide). Hypoglycemic episodes occurred in three patients (12.5%), all of whom were receiving concomitant basal insulin. Mild urinary tract infections were observed in four patients (17%), with no cases requiring discontinuation of therapy. No cases of pancreatitis, gallbladder disease, or other serious adverse events were reported.

**Table 4:** Changes in clinical parameters from baseline to follow-up during GLP-1 RA therapy in KTRs with PTDM

Variable	Baseline	Follow-up	∆	p-Value
	mean ± SD	mean ± SD	mean ± SD	
Glycemic control HbA1c (%) FPG (mg/dL)	7.4 ± 1.7 114.5 ± 29.8	7.2 ± 1.2 126.5 ± 37.9	0.2 ± 1.4 +12.0 ± 48.3	0.362 0.232
Lipid panel Total cholesterol (mg/dL) LDL-c (mg/dL) HDL-c (mg/dL) Triglyceride (mg/dL)	182.9 ± 61.9 94.9 ± 51.7 49.5 ± 11.9 200.5 ± 126.9	141.4 ± 32.6 72.4 ± 28.6 47.9 ± 10.6 166.9 ± 70.8	-41.5 ± 47.9 -22.5 ± 41.3 -1.6 ± 9.3 -33.6 ± 110.3	**0.0003** **0.014** 0.399 0.149
Anthropometric measures Body weight (kg) BMI (Kg/m^2^)	73.2 ± 14.7 27.3 ± 5.4	69.6 ± 14.0 25.9 ± 5.2	--3.6 ± 8.5 -1.4 ± 3.3	0.051 0.056
Hemodynamic data Systolic BP (mmHg) Diastolic BP (mmHg)	143.0 ± 15.7 71.5 ± 18.3	134.5 ± 15.6 75.0 ± 13.0	-8.5 ± 19.3 +3.5 ± 14.9	**0.041** 0.665
Renal function Creatinine (mg/dL) eGFR (mL/min/1.73 m^2^)	1.7 ± 0.6 46.1 ± 15.8	1.6 ± 0.6 49.3 ± 14.7	-0.1 ± 0.4 +3.2 ± 11.9	0.305 0.206

BP: blood pressure; BMI: body mass index; eGFR: estimated glomerular filtration rate; FPG: fasting plasma glucose; HbA1c: glycated hemoglobin; KT: kidney transplantation; PTDM: post-Transplant diabetes mellitus; SD: standard deviation.

## DISCUSSION

In this retrospective study of 24 KTRs with PTDM, add-on therapy with GLP-1 RAs over a mean follow-up of 3.2 years was associated with significant improvements in total and LDL cholesterol and SBP, as well as non-significant trends toward weight and BMI reduction. Glycemic control, assessed by HbA1c and FPG, remained stable throughout the follow-up, and renal function was preserved. These findings contribute to the growing body of evidence regarding the potential role of GLP-1 RAs in the post-transplant setting.

In our cohort, the modest HbA1c reduction of 0.2% is consistent with the variable glycemic responses reported in the literature. Kukla and cols. (^20^) reported a mean reduction of 0.6% in a heterogeneous cohort consisting of both kidney and multi-organ transplant recipients with either pre-existing diabetes or PTDM. Singh and cols. (^21^) observed a mean reduction of 0.7% after six months in a mixed transplant population, while Mallik and cols. documented a 1.3% decrease in KTRs (^22^). A more pronounced reduction of 1.9% was described by Liou and cols. (^23^) over 19.4 months in a small retrospective series. In contrast, Kim and cols. (^24^) found no significant change when switching from prandial insulin to dulaglutide. This variation underscores the heterogeneity of treatment response across different study populations and settings.

Unlike the REWIND trial (^14^), in which dulaglutide was associated with modest reductions in LDL-c and total cholesterol, our predominantly dulaglutide-treated cohort exhibited more pronounced declines in both parameters. We also observed non-significant improvements in HDL-c and triglycerides. These lipid outcomes are consistent with the diverse effects reported in other cardiovascular outcome trials: LEADER (^11^) and SUSTAIN-6 (^12^) demonstrated reductions in LDL-c and triglycerides without significant changes in HDL-c, while PIONEER 6 reported minimal lipid alterations (^13^).

Similar benefits were noted in blood pressure, with a clinically meaningful reduction in SBP (8.5 mmHg), exceeding the modest declines reported in those trials, where systolic reductions varied by 1.2-2.6 mmHg (^11^,^12^,^14^), whereas DBP remained stable. These findings reinforce the potential hemodynamic advantages of GLP-1 RAs, particularly in high-risk transplant recipients. Beyond lipid and blood pressure improvements, GLP-1 RA therapy was associated with a trend toward weight reduction (mean loss of 3.6 kg; *p* = 0.051) and a decrease in BMI (1.4 kg/m^2^; *p* = 0.056). Although these changes did not reach statistical significance, they are in line with earlier studies among transplant populations. Mallik and cols. reported a mean weight reduction of 2.7 kg and a BMI decrease of 0.9 kg/m^2^ after one year of therapy (^22^), while Kukla and cols. (^20^) observed a greater reduction of 9.4 kg and 1.9 kg/m^2^. Collectively, these findings suggest a possible role for GLP-1 RAs in mitigating post-transplant weight gain, which is a key contributor to metabolic dysfunction in this population.

Although our study was not designed to assess cardiovascular outcomes, the observed improvements in body weight, blood pressure, and lipid profile suggest a potential for cardiometabolic risk modification. This is particularly pertinent for transplant recipients, in whom obesity reflects not only excess adiposity, but also chronic metabolic dysfunction and persistent systemic inflammation-both established contributors to cardiovascular morbidity. The potential cardioprotective value of GLP-1 RAs is supported by large-scale outcome trials demonstrating significant cardiovascular benefits: LEADER reported a 13% reduction in MACEs with liraglutide, primarily due to decreased cardiovascular mortality (^11^); SUSTAIN-6 showed a 26% MACE reduction with semaglutide, largely attributable to reduced stroke risk (^12^); and REWIND demonstrated a 12% MACE reduction with dulaglutide, even in a primary prevention cohort (^14^). Considered alongside the favorable metabolic profile observed in our cohort, these results highlight the potential role of GLP-1 RAs in reducing cardiovascular risk among KTRs with PTDM.

Regarding renal safety, graft function remained stable throughout follow-up, with no significant changes in serum creatinine or eGFR. While statistically neutral, this finding may be clinically relevant, as KTRs typically experience progressive graft function decline-a trend not observed in our cohort. These results are consistent with previous reports in transplant populations (^20^,^25^), in which GLP-1 RA therapy was also associated with stable renal function. Additionally, tacrolimus trough levels remained stable in the 20 patients with available data, suggesting no clinically relevant drug interaction or absorption changes with GLP-1 RAs, despite theoretical concerns related to delayed gastric emptying. Nevertheless, given the small sample size and lack of a comparator group, both renal function and tacrolimus findings should be interpreted with caution, as studies of GLP-1 RA safety in KTRs remain limited.

GLP-1 RA therapy was generally well tolerated. Treatment discontinuation occurred in 17% of patients (4/24), all due to gastrointestinal intolerance-mainly nausea-while most patients maintained long-term therapy. Hypoglycemic events occurred exclusively in patients receiving concomitant insulin (3/24; 12.5%), and no cases of pancreatitis, gallbladder disease, or hypersensitivity reactions were observed. These findings reinforce the favorable safety and tolerability profile of GLP-1 RAs in KTRs when accompanied by appropriate monitoring and individualized dosing.

### Study limitations

This study has several limitations inherent to its retrospective, single-center design, including small sample size (n = 24), absence of a control group, and potential unmeasured confounders (e.g., treatment interruptions and lifestyle factors. The inclusion of three different GLP-1 RAs (i.e., dulaglutide, liraglutide, and semaglutide) limits our ability to assess agent-specific effects, as these agents vary in potency, weight-loss efficacy, cardiovascular benefit, and gastrointestinal tolerability. Pooling them may therefore obscure differences in their therapeutic profiles. The exclusively White cohort further restricts generalizability to more diverse populations, and the concomitant use of other antidiabetic agents, including insulin and, in four cases, DPP-4 inhibitors, precludes definitive attribution of observed effects to GLP-1 RA therapy alone. Although no changes in antihypertensive drug classes were noted, changes in dosage of existing medications during follow-up were not systematically recorded; this represents an unquantifiable confounder in assessing the isolated hemodynamic effects of GLP-1 RAs. Additionally, the mean interval of 7.6 years from transplantation to GLP-1 RA initiation introduces a survivor bias, as patients must have survived and maintained graft function long enough to receive this therapy, potentially selecting for a metabolically stable, adherent, and healthier subgroup, and limiting generalizability to the broader PTDM population. Despite these limitations, the study has notable strengths, including a prolonged mean follow-up of 3.2 years (considerably longer than most previous reports), allowing assessment of sustained treatment effects. The homogeneous cohort of exclusively KTRs with PTDM enhances clinical relevance, while the inclusion of multiple GLP-1 RA agents and diverse background regimens reflects real-world clinical complexity and supports the applicability of our findings to routine practice.

In conclusion, this study demonstrated that GLP-1 RA therapy in KTRs with PTDM is associated with significant improvements in total cholesterol and SBP, along with favorable trends in weight reduction and stable renal function over a mean follow-up of 3.2 years. Glycemic control remained unchanged, and the safety profile was acceptable, with gastrointestinal intolerance identified as the most common adverse event. Although these findings support the potential role of GLP-1 RAs in managing this high-risk population, the modest effects on glycemic control and the non-significant weight loss observed underscore the need for larger, prospective controlled studies to confirm long-term cardiorenal benefits and the optimal of these agents in the post-transplant setting.

## Data Availability

datasets related to this article will be available upon request to the corresponding author.
